# Advances in Leaf Plant Bioactive Compounds: Modulation of Chronic Inflammation Related to Obesity

**DOI:** 10.3390/ijms26073358

**Published:** 2025-04-03

**Authors:** Jorge Barros, Ana Abraão, Irene Gouvinhas, Daniel Granato, Ana Novo Barros

**Affiliations:** 1 Centre for Research and Technology of Agro-Environmental and Biological Sciences, CITAB, Inov4Agro, University of Trás-os-Montes and Alto Douro, UTAD, Quinta de Prados, 5000-801 Vila Real, Portugal; aabraao@utad.pt (A.A.); igouvinhas@utad.pt (I.G.); 2 Department of Agricultural sciences, Higher Polytechnic Institute of Bengo, B. Caboxa, Dande, Bengo 244-2004, Angola; 3 Bioactivity & Applications Laboratory, Department of Biological Sciences, Faculty of Science and Engineering, School of Natural Sciences, University of Limerick, V94 T9PX Limerick, Ireland; daniel.granato@ul.ie

**Keywords:** obesity, chronic inflammation and oxidative stress, bioactive compounds, leaves, anti-obesity capacity

## Abstract

Over the years, there has been a tendency for an increase in global obesity. The World Health Organization’s (WHO) 2024 report states that in 2019, more than one billion people were obese, and this condition was responsible for five million deaths, being that obesity is more prevalent among adults compared to adolescents and children. Obesity is a chronic disease characterized by alterations in adipose tissue. When excessive food is consumed and energy expenditure is low, adipose tissue undergoes hypertrophy and hyperplasia. This process activates B cells and induces the transition of anti-inflammatory M2-like macrophages into pro-inflammatory M1-like macrophages. B cells, acting as inflammatory mediators, stimulate pro-inflammatory CD8+ T cells, and promote macrophage infiltration into tissues. This condition triggers inflammation, increases oxidative stress, and ultimately leads to cellular death. During inflammation, an increase of pro-inflammatory cytokines occurs along with a decrease of anti-inflammatory cytokines. By contrast, the increase of oxidative stress is related to an increase of reactive oxygen species (ROS), oxidation of biomolecules, and a decrease in antioxidants. This mechanism for obesity can be mitigated through several healthy lifestyle changes, primarily including regular physical activity and healthy eating. These factors help reduce pro-inflammatory mediators and ROS, lowering inflammation and oxidative stress. Therefore, this review article focuses on studying the bioactive compounds present in the edible leaves of *Annona cherimola* Mill., *Ipomoea batata* (L.) Poir., *Colocasia esculenta* (L.) Schott, *Eriobotrya japonica*, *Cymbopogon citratus*, *Psidium guajava* (L.), and *Smallanthus sonchifolius* to evaluate their effects on the mechanisms involved in obesity.

## 1. Introduction

In general terms, obesity is a medical condition that can arise from heart disease and multiple other health complications, including type 2 diabetes (T2DM). It is marked by low-grade chronic inflammation and results from multiple factors, including behavioral patterns like eating, sleeping, exercising, and taking medicines, as well as genetic influences and family history [1].

As per the World Health Organization (WHO), obesity is primarily regarded as an issue for public health along with a significant prevalence of comorbidities, such as metabolic, cardiovascular, respiratory, osteoarticular, gastrointestinal, endocrine, psychological diseases, and even cancer, occurring at the same time or as a result of increased fat mass, which affect nearly 25% of the global population [2]. This disease is described as excessive body fat in individuals who are afflicted with a disproportionate amount of high body fat together with an increase in the body’s lipid tissues [1,3]. The adipose tissues usually increase due to an expansion of adipocytes, which is called hypertrophy, or during the increase of adipocytes, called hyperplasia of fat cells [1,4]. Obesity has many indicators, one of them being the body mass index (BMI), which calculates the ratio of a person’s weight to their height, considering and measuring in kilograms per square meter. In this case, obesity is the condition when the BMI is greater than 30 kg/m^2^ [4]. Obesity can be classified using BMI according to its severity into grade I (when the BMI reaches 35 kg/m^2^), grade II (when the BMI is equal to or less than 39.9 kg/m^2^), and grade III (when the BMI is greater than 40 kg/m^2^).

An increase in an individual’s BMI reflects increases in the proportion of body fat and, consequently, in the mortality rate. Globally, the proportion of adults with a high BMI has increased substantially in both developed and developing countries, making obesity a major global health challenge. In Europe, in 2016, it was estimated that adults aged 50 and over represented almost 40% of the population [5].

The WHO reported that, in 2019, 5 million deaths were due to non-communicable diseases (NCDs) caused by a greater than optimal BMI. Rates of obesity have continually increased among adults and children between 1990 and 2022, rising globally from 2% to 8% for those aged 5–19 years and from 7% to 16% for those over the age of 18. The 2015/2016 Report *Obesity in the European Region* reports that over 20% of Portugal’s population had an above average BMI, i.e., six in ten Portuguese adults were overweight and obese; with the Azores showing the greatest prevalence (32.9%), followed by Alentejo (27.6%), while Centre had the lowest rates (19%), as well as the North at 21.5%. Pre-obesity was highest in Madeira (37.0%) and Algarve (37.3%) according to results recorded in [6,7,8].

Recently, there has been a significant increase in measures to monitor changes in the prevalence of obesity in all populations. Tracking the prevalence and trends of obesity is crucial for evaluating interventions aimed at preventing or reducing the burden of obesity [9,10]. One of the alternatives to prevent obesity as a risk factor for cardiometabolic diseases may be the adoption of a healthier lifestyle and eating habits [10]. However, interest in the expansion of anti-inflammatory and anti-obesity drugs from natural sources, such as plants and their by-products, that are rich in bioactive compounds, including polyphenols, has increased considerably [11].

Polyphenols are natural compounds present in plants with numerous biological activities that have been widely studied for their ability to eliminate endogenously generated free radicals [12]. Their anti-inflammatory, anti-obesity, and antioxidant properties are mediated mainly by the ability to downregulate the nuclear factor kappa B (NF-kB), modulating crucial cell signaling pathways involved in inflammation [13], and switching between macrophages of M1 and M2 phenotypes [14]. This immune-modulating effect of polyphenols is supported by the modulation of immune cell populations, cytokine production, and the expression of pro-inflammatory genes [15].

Recent studies have considered that, on average, there is a global trend towards low consumption of fruits and vegetables and high consumption of fast food and sugary drinks, especially in industrialized countries [16]. Therefore, in this study, we propose a literature review focused on the phenolic compounds present in the leaves of *Annona cherimola* Mill., *Ipomoea potato* (L.) Poir., *Colocasia esculenta* (L.) Schott, *Eriobotrya japonica*, *Cymbopogon citratus*, *Psidium guajava* (L.), and *Smallanthus sonchifolius* to modulate chronic inflammation associated with obesity, from its bioactivity in the inflammatory and oxidative mechanisms.

## 2. Chronic Inflammation and Oxidative Stress Associated with Obesity

**Figure 1:**
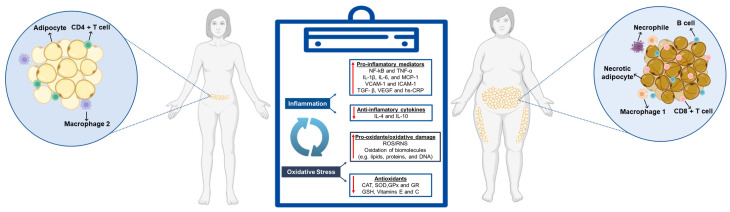
Mechanism of obesity. CAT (catalase); SOD (superoxide dismutase); GPx (glutathione peroxidase); GR (glutathione reductase); GSH (glutathione); vitamin E (tocopherol); vitamin C (ascorbic acid); NF-κB (nuclear factor kappa B); TNF-α (tumor necrosis factor-alpha); IL-1β (interleukin-1 beta); IL-6 (interleukin-6); MCP-1 (monocyte chemoattractant protein-1, also known as CCL2); VCAM-1 (vascular cell adhesion molecule-1); ICAM-1 (intercellular adhesion molecule-1); TGF-β (transforming growth factor-beta); VEGF (vascular endothelial growth factor); hs-CRP (high-sensitivity C-reactive protein); ROS (reactive oxygen species) and RNS (reactive nitrogen species); IL-4 (interleukin-4) and IL-10 (interleukin-10).

Chronic inflammation lasts for a prolonged period and is marked by lymphocytes, macrophages, as well as the growth of new blood vessels/tissues [17,18]. In obesity, a low-grade inflammation exists, which causes metabolic disorders, like insulin resistance (IR), T2DM, overweight issues, hypertension, heart disease, abnormal levels of lipids in the blood, and cancer [1]. Increased macrophage aggregates indicate the inflammatory condition of obesity. In obese individuals, macrophages switch from an M2 (anti-inflammatory) to an M1 state (pro-inflammatory), which is tightly associated with insulin resistance, possibly because of inflammation in the adipose tissue. This includes alterations, such as adipose tissue fibrosis, B cell-derived cytokines, and infiltration of macrophages into fat tissue, which consequently overproduce pro-inflammatory mediators. These mediators, or biomarkers, locally initiate inflammation that subsequently becomes systemically allied with the onset of obesity-induced co-morbidities [19]. In this way, macrophages are also known to produce various inflammation-related biomarkers, like tumor necrosis factor-alpha (TNF-α), interleukin six (IL-6), and adiponectin. IL-6 also strongly induces the synthesis and secretion of C-reactive protein (CRP) by hepatocytes, thus reflecting an inflammatory state [17,18]. Studies have shown a positive correlation between obesity indices and inflammatory biomarkers, most notably CRP in females; however, other inflammatory markers were also found to be associated with both sexes [20], as depicted in Figure 1.

Obesity’s low-grade chronic inflammation significantly causes oxidative stress as well. It is linked to increased plasma leptin levels that, as a hormone, activate NADPH oxidase and induce the production of reactive intermediates, including hydrogen peroxide (H_2_O_2_) and hydroxyl radical (^·^OH). Both TNF-α and IL-6 increase the activities of reactive nitrogen species (RNS) and the production of superoxide anion (O_2_^•─^) with increased electron interaction with oxygen [21]. RNS are essential signaling molecules that, when uncontrolled, can cause damage to cellular proteins, lipids, and DNA, with detrimental effects on functions. High levels of circulating glucose and lipids increase the energy substrates delivered to metabolic pathways in obesity, and can enhance mitochondrial dysfunction and RNS production [22].

### 2.1. Inflammatory Biomarkers: Mechanisms and Health Impacts

Low-grade chronic systemic inflammation, regarded as the result of weight gain and fat tissue accumulation, particularly abdominal fat, is characterized by the increased serum levels of inflammatory markers, such as IL-6, interleukin 1β (IL-1β), and TNF-α, among others, as well as inflammatory biomarkers and blood markers indicating inflammatory reaction, such as CRP, leukocyte count, and fibrinogen [22,23]. This is associated with the fact that the fat tissue inflammation induces the loss of immune modulation as well as the infiltration and activation of immune cells which, in turn, leads to increased production of pro-inflammatory cytokines, such as IL-6, Interleukin 1α (IL-1α), IL-1β, TNF-α, plasminogen activator inhibitor-1 (PAI-1), and monocyte chemoattractant protein-1 (MCP-1) [24]. The serum levels of inflammatory biomarkers, such as TNF-α, CRP, IL-6, and IL-18, are changed in overweight and obese people [19]. The primary inflammatory molecule characteristic of systemic inflammation, CRP, is produced and secreted by the liver when TNF-α is overexpressed in the overweight state, whereas IL-6 is more frequently linked to the obese state [17,18]. Pro-inflammatory signaling pathways in adipose tissue macrophages trigger the cytokines TNF-α and IL-6, which are increased in obese people. Because pro-inflammatory macrophage pathways upregulate the chemokine MCP-1, pro-inflammatory immune cells can more readily penetrate adipose tissue [19,25]. Adipocytes and immune cells found in the enlarged adipose tissue, including fibroblasts, endothelial cells, and immune system cells, are the primary sources of inflammatory cytokines. These cells can release chemokines and inflammatory cytokines. Visceral adipose tissue secretes more TNF-α and ILs than subcutaneous abdominal adipose tissue [22], as represented in Figure 1.

#### 2.1.1. Tumor Necrosis Factor-Alpha (TNF-α)

TNF-α is produced by several cells, such as macrophages, lymphoid cells, endothelial cells, cardiac myocytes, adipose tissue, and brain cells (e.g., microglia and astrocytes) [26]. It plays a role in the pathogenesis of IR and T2DM. It also reduces the expression of the insulin-regulated glucose transporter type 4 (GLUT4), primarily localized in adipocytes, as well as in skeletal and cardiac muscles [27]. Tabling the issue of obesity, it becomes apparent that higher levels of TNF-α, in obesity, result from increased macrophage infiltration into adipose tissue cells. TNF-α plays a different role in obesity, and it can cause an increase in a person’s glucose resistance and obesity/T2DM, a change in adipogenesis, and lipolysis, thus increasing other cytokines production (such as MCP-1, IL-6) which, in turn, will aggravate systemic inflammation [28]. This process is initiated with the activation of free fatty acids in adipocytes, which leads to the inhibition of adiponectin synthesis, an insulin sensitizer, that is abundant in adipose tissue. Additionally, the phosphorylation activity of the insulin receptor substrate 1 (IRS-1) at the tyrosine residue, which is essential for intracellular signaling, becomes disrupted [29]. It triggers NF-κB, causing adhesion molecules to be overexpressed on endothelial cells and vascular smooth muscle cells’ surfaces, leading to inflammation within adipose tissue, endothelial dysfunction, and eventually atherogenesis [29,30]. It increases PAI-1 in adipocytes via the TNF-α signaling pathway linked to TNF receptor 1 (TNFR1), involving the activation of NF-κB production, reactive oxygen species (ROS), and increased inflammation [24]. It regulates liver homeostasis, including hepatocyte proliferation. Nevertheless, apoptosis and necroptosis in hepatocytes are regulated. Once TNF-α is released, it adheres to TNFR1 or TNF receptor 2 (TNFR2). The primary signaling occurs through TNFR1, as the TNFR1 by signaling complex I causes activation of the pro-inflammatory NF-κB and mitogen activated protein (MAP) kinase pathways, IκB kinase (IKK) and NF-κB activate TNF-α in fat, so obese individuals’ adipocytes, when directly exposed to TNF-α become hyper-responsive and secrete large number of adipokines [31]. TNFR1 and the NF-κB pathway hyperactivation mediate this hyper-responsiveness. Accordingly, in adipocytes from obese individuals, NF-κB activity is increased and correlates with adipocyte size and adipokine expression as well as in vivo insulin resistance. As a result, NF-κB inhibitors eventually prevent the overexpression of adipokines in obese adipocytes [32].

#### 2.1.2. Interleukin 6 (IL-6)

IL-6 is a key cytokine released by macrophages and adipocytes (e.g., skeletal muscle, fibroblasts, and endothelial cells) in response to immune reaction. Other cytokines, primarily interleukin-1 (IL-1) and TNF-α, also induce its secretion [33,34]. Yet, studies have shown that adipose tissue is responsible for about 35% of circulating basal IL-6 in humans [35,36]. IL-6 systemically regulates body weight and lipid metabolism, and it is also involved in obesity, as well as IR [34]. In obesity, chronically elevated IL-6 levels may exert beneficial or pathogenic effects on energy metabolism across multiple organs, including the liver, pancreas, and adipose tissue [36]. When it functions away from where it was produced, this circulating cytokine acting via an endocrine mechanism is hence referred to as an endocrine cytokine [18,36]. It is involved in everything, from immune defense to inflammation and tissue repair. Its levels in the blood are linked to BMI, insulin resistance, and carbohydrate intolerance. It also modulates glucose tolerance (GT) by decreasing visfatin and counteracting adiponectin secretion [29]. Both TNF-α and IL-1 are expressed in the hypothalamus and other regions of the brain that are responsible for energy homeostasis through the inhibition of lipoprotein lipase activity, appetite, and energy expenditure [17,31]. To activate the classical signaling, the glycoprotein 130 (gp130) homodimerized signal transducing receptor immediately condenses with IL-6 that has already bound its unique receptor (interleukin 6 receptor IL-6Ra), which is situated on the cell membrane. IL-6Ra is used not only by IL-6 but by ciliary neurotrophic factor (CNTF). However, all remaining cytokines of the type IL-6 are dependent on gp130 to trigger intracellular signaling, as this receptor is present in every living cell, whereas a membrane attached IL-6Ra receptor is restricted only to hepatocytes, epithelial cells, and leukocytes [35].

#### 2.1.3. C-Reactive Protein (CRP)

CRP is an acute inflammatory protein synthesized in hepatocytes under the primary stimulus of IL-6 [33], reflecting systemic inflammation, which is a component of the state of obesity, T2DM, and atherosclerosis, likely due to cytokine stimulation by adipose tissue, among others [37,38]. When elevated for prolonged periods, it is a characteristic of acute inflammation due to ongoing inflammation [37]. It typically increases in the first six to eight hours in acute inflammatory conditions, and its values may be as high as 300 mg/dL by 48 h [33]. CRP values of 3 mg/L indicate a high relative risk [39]. Its upregulation is a downstream consequence of IL1-mediated pro-inflammatory signaling by the IL-6 receptor complex through the NLR family pyrin domain containing 3 (NLRP3) inflammasome [38]. CRP is a highly sensitive protein found in the blood due to inflammation, leading to an increased possibility of cardiovascular disease and atherosclerosis. This condition also correlates with other classical risk factors, such as creatine kinase (CK) and lactate dehydrogenase (LDH) [30,33,38,40]. It promotes the adhesion-regulating endothelin ET-1, causing the entry of low-density lipoprotein cholesterol (LDL) into macrophages through MCP-1. CRP also controls NF-κB, which aids many pro-atherosclerotic genes, acts at vasodilatory prostacyclins, which are produced in lesser quantity, and causes destabilization of the fibrous cap of the atheroma by stimulating matrix metalloproteinase-1 (MMP-1) which is released during collagen and protein destruction, fibrinolysis is declined, and PAI-1 is increased. CRP further acts as a mediator in the formation of atherosclerotic plaque through its participation in the inhibition of complement mediator proteins [33].

## 3. Importance of Dietary Interventions in Managing Chronic Inflammation/Oxidative Stress Related to Excessive Weight and Obesity

Dietary interventions are crucial in managing chronic inflammation and oxidative stress associated with excessive weight (Table 1). While the involvement of diet in inflammation is being actively studied, a strong link between nutrition and immune system function, along with inflammatory response, has been established [41]. Mediterranean diets, diets high in fruits, vegetables, other plant foods, and fiber-rich diets (including supplementation) have been shown to reduce chronic inflammation and oxidative stress [42]. On the other hand, carbohydrate-heavy meals loaded with refined grains, sugars, and sugary drinks, increase inflammatory markers in normal weight and overweight individuals. They may also acutely raise blood glucose levels for a short period to induce pro-inflammatory cytokines like IL-6, IL-18, and TNF-α. Moreover, the intake of saturated fats and *trans* fats modifies the amount and type of macronutrients consumed, which may lead to increased CRP levels [43].

Consuming foods rich in antioxidants and polyphenols increases the plasma antioxidant capacity and reduces oxidative stress markers and chronic inflammation among people suffering from metabolic syndrome or related disorders. Weight loss, by itself, can improve antioxidant capacity, but when combined with large amounts of antioxidants in low-calorie diets, this is synergistic [44].

Overall, the results in Table 1 indicate that healthy diets, such as the Mediterranean diets and other anti-inflammatory diets, have a protective effect against inflammation, as they are associated with the modulation and reduction of inflammatory markers, such as TNF-α, IL-6, and CRP [45]. Vegetarian and vegan diets are related to low concentrations of CRP [46] and anti-inflammatory diets with caloric restriction, highlighting the importance of dietary intervention in controlling inflammation in obese individuals, which improves inflammatory parameters (reduction of TNF-α, CRP, and IL-6), in addition to promoting weight and visceral fat reduction [47]. On the other hand, another study suggests that high-fat meals induce the inflammatory response, increasing IL-6 levels without affecting CRP and TNF-α [48]. However, sugar-sweetened beverage (SSB) intake increases adiposity and the inflammatory profile, with an increase in the concentrations of pro-inflammatory markers, such as leptin, CRP, and MCP-1 [49,50,51].

**Table 1:** Summary of current evidence on the effect of major dietary patterns and food groups on inflammatory biomarker levels.

**References**	**Objective/Methods**	**Target Audience**	**Study Type**	**Results**
[45]	To evaluate the associations between dietary patterns and one or more inflammatory biomarkers (CRP, TNF-α, and IL-6).	Healthy women and men aged 18 years or older	Meta-Analyses (PRISMA).	Healthy, Mediterranean, and anti-inflammatory diets are most frequently associated with lower levels of inflammation (reduction in CRP, TNF-α, and IL-6).
[48]	To characterize the postprandial response of 5 commonly assessed inflammation markers after ingesting a high-fat meal (HFM) in healthy adults.	Men or women aged between 18 and 60 years divided into: Healthy individuals without a diagnosis of chronic disease. Overweight and obese individuals without any other associated chronic disease.	Meta-Analyses and cohort.	Only 1 of these 5 markers, IL-6, consistently increases in the post-HFM period of 4 to 8 h. Specifically, IL-6 will start on average, at a baseline of approximately 1.4 pg/mL and peak at approximately 2.9 pg/mL about 6 h later. In relative terms, IL-6 will increase by approximately 100% in response to an HFM. As for CRP and TNF-α, these rarely show any change. IL-8 and IL-1β also rarely change after HFM consumption in healthy individuals.
[52]	To evaluate the association of a diet high in SSB and low in fruits and vegetables with adiposity and a pro-inflammatory adipokine profile.	Mexican-American participants ascendent, with and without a diagnosis of gestational diabetes mellitus (GDM) within 5 years.	Fenland Study.	A diet high in SSB and low in fruits and vegetables may be associated with increased adiposity and a pro-inflammatory adipokine profile characterized by higher leptin, CRP, and MCP-1, and lower anti-inflammatory secreted frizzled-related protein 5 (SFRP-5) in Mexican Americans compared to a diet low in SSB and high in fruits and vegetables.
[49]	To evaluate the increase in plasma uric acid levels in overweight and obese individuals consuming sucrose-sweetened sodas for 6 months	60 eligible participants with overweight and obesity without diabetes.	Meta-Analyses and Randomized Controlled Trials (RCT).	Daily 1 L of sugar-sweetened soft drinks (regular cola/sucrose-sweetened soda) for 6 months increases circulating UA levels. Additionally, it has been demonstrated that changes in plasma UA after the intervention significantly correlate with changes in liver fat levels, triglycerides, and insulin.
[50]	To analyze the cross-sectional association between SSB intake and cardiometabolic biomarkers in American women.	121,700 registered nurses aged between 30 and 55 years, free of diabetes and cardiovascular disease (CVD).	Fenland Study.	The intake of SSB was marginally associated with higher concentrations of CRP and adiponectin (suggesting liver function, lipid metabolism, inflammation, and glucose metabolism as possible pathways). The associations between artificially sweetened beverages (ASB) and fruit juice with cardiometabolic markers were less consistent.
[53]	To examine the association of sugars from different sources [beverages (liquids), foods (solids), extrinsic (free) or intrinsic (non-free)] with metabolic and inflammatory markers.	12,434 individuals born between 1950 and 1975, recruited from general practitioner lists in Cambridgeshire and surrounding areas in the East of England, UK, enrolled between 2005 and 2015.	Fenland Study.	Higher intakes of sugars from non-alcoholic beverages and sugar added to tea, coffee, or cereals were associated with increased blood glucose and CRP.
[51]	To evaluate the associations between SSB consumption and CRP levels; To evaluate the modifying effect of BMI on the association between SSB consumption and CRP levels.	6856 American adults from the National Health and Nutrition Examination Survey (NHANES) 2007–2010. The average SSB consumption was calculated from 2-day and 24-h dietary recalls.	Fenland Study.	The intake of SSB is positively associated with CRP levels. Obesity may amplify CRP levels in individuals with moderate to high SSB consumption.
[39]	To investigate the effect of increased dietary intake of alpha-linolenic acid (ALA) on blood concentration of inflammatory markers, including TNF-α, IL-6, CRP, soluble intercellular adhesion molecule-1 (sICAM-1), and soluble vascular cell adhesion molecule-1 (sVCAM-1).	Men and women, adults, both with and without obesity.	Meta-Analyses.	The study found no beneficial effect of ALA supplementation in reducing inflammatory markers, including TNF, IL-6, CRP, sICAM-1, and sVCAM-1. However, in healthy individuals, ALA supplementation may increase CRP concentration.
[46]	To evaluate the association of vegan and vegetarian diets with inflammatory biomarkers.	Adult individuals with different dietary habits, ranging from apparently healthy vegans, vegetarians, and omnivores to those with metabolic issues.	Meta-Analyses (PRISMA) included cross-sectional studies, prospective cohort studies, and RCT.	The vegan and vegetarian diets are associated with lower CRP concentrations compared to apparently healthy omnivores and others with metabolic issues.
[54]	To examine the associations of dietary macronutrient composition in early childhood with growth and detailed measures of body composition up to 9 years of age.	3564 Dutch children aged 1 to 9 years.	Cohort Study.	Higher intake of total and animal protein (dairy and non-dairy) is associated with greater height, weight, and BMI up to 9 years of age. The positive association with BMI was fully explained by a higher fat mass index (FMI) and not by the fat-free mass index (FFMI).
[47]	To determine changes in body composition and cardiometabolic and inflammatory status of obese participants after 24 weeks of a dietary intervention based on an anti-inflammatory diet with energy reduction and examine the relationship of these changes with changes in the inflammatory potential of the diet.	In the past three months, adult male and female patients from the Clinical Hospital Center Rijeka, Croatia, with BMI ≥ 30 kg/m^2^, with or without obesity-related complications, and stable body weight.	RCT Study	An anti-inflammatory diet with energy restriction is effective in managing obesity (resulting in high reductions of TNF-α and low levels of hs-CRP and IL-6). Significant reductions in body weight, BMI, total and visceral adipose tissue, and improvements in body composition, cardiometabolic parameters, and inflammatory markers were observed.

## 4. Bioactive Compounds in Leafy Vegetables

Currently, there is a strong focus on broadening the scope of anti-inflammatory and anti-obesity treatments, especially considering natural sources, such as plants [11]. Through photosynthesis, which serves as the primary synthesis tool for organic compounds, plants, the most efficient living organisms, exhibit incredible molecular diversity [55]. All these compounds are, to a great extent, dependent on the basic phytochemical constituents which, in turn, depend on their basic metabolic processes or metabolic pathways, including primary and secondary metabolites [56]. Secondary metabolites are organic compounds that are usually produced as a result of the alteration of primary metabolites, which form part of the secondary metabolic pathway [57]. These compounds, which can be classified as bioactive compounds and exhibit a wide range of effects on biological systems, are produced in extremely low concentrations within plant cells, and rely significantly on the physiological stage and development of the plant. Hence, bioactive compounds derived from plants and classified as secondary metabolites are deemed to be associated with the pharmacological and/or toxicological effects they inflict on humans and/or animals [58].

**Figure 2:**
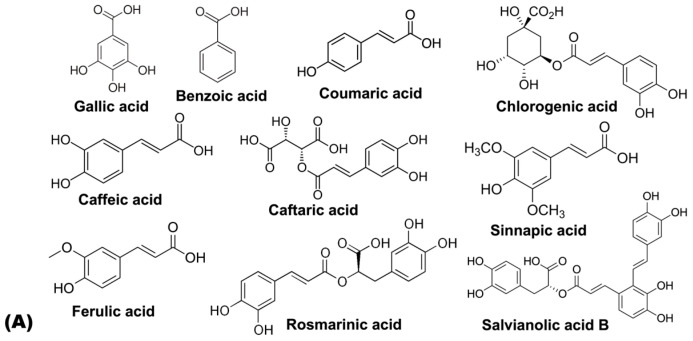

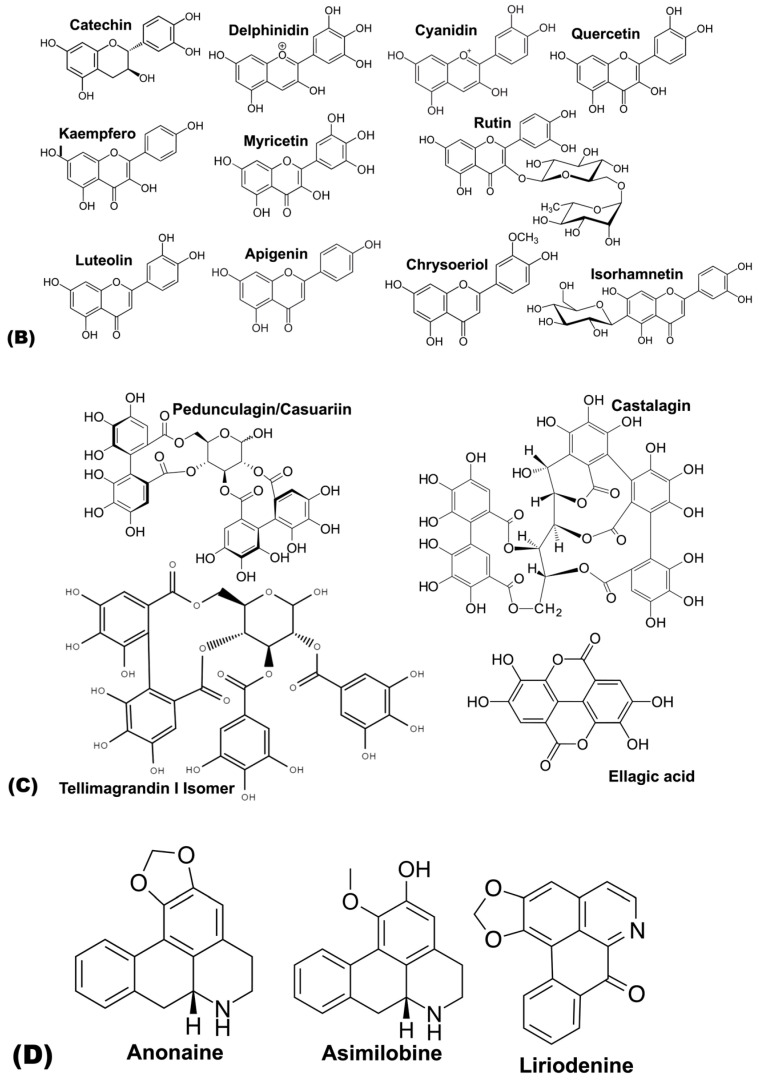
Chemical structure of the main compounds identified in the leaves of the species under study as: (**A**) phenolic acids like hydroxybenzoic acids and hydroxycinnamic acids; (**B**) flavonoids like flavanols, flavonols, and flavones; (**C**) hydrolysable tannins; and (**D**) alkaloids.

The classification of bioactive compounds is not unanimous and relies upon the authors [57]. They can be classified in different ways, including based on structure, as depicted in Figure 2, composition, solubility in various solvents, and synthetic pathways. Bioactive compounds can be classified, depending on their structures, into multiple classes and subclasses. Phenolic compounds include coumarins, stilbenes, phenolic acids (subdivided into hydroxycinnamic and hydroxybenzoic acids), and flavonoids, like flavonols, flavones, flavanols, flavanones, anthocyanins, and isoflavones. Additionally, other bioactive compounds include terpenoids (monoterpenes, sesquiterpenes, diterpenes, sesterterpenes, triterpenes, tetraterpenes, and polyterpenes), tocopherols, and carotenoids. Nitrogenous compounds, such as alkaloids and cyanogenic glycosides, as well as sulfur-containing compounds, including glutathiones, glucosinolates, and thiocompounds, are also biologically relevant [56]. Vitamin C and fiber are widespread, generalized phytochemicals.

Common phenolic compound classes with biological roles present in food include flavonoids, phenolic acids, stilbenes, and lignans. Such compounds, when included as part of a diet, affect human health by exhibiting antioxidant, antimicrobial, anti-inflammatory, vasodilatory, and prebiotic effects [4].

Table 2 indicates that phenolic compounds occur extensively in the leaves of the plant species addressed in this review, and fall within several main chemical categories, which include specific sub-classes: phenolic acids (hydroxybenzoic acids and hydroxycinnamic acids), flavonoids (flavanols, flavonols, flavones), hydrolysable tannins, and alkaloids; specifically: (i) Hydroxybenzoic acids, such as gallic acid, were identified in *Cymbopogon citrates*, *Psidium guajava* (L.), and *Smallanthus sonchifolius*, while benzoic acid was found in *Cymbopogon citratus*. (ii) Hydroxycinnamic acids, like chlorogenic acid and its derivatives were noted in nearly all the plants; caffeic acid and its conjugates (caffeoylquinic) were commonly found, except in *Annona cherimola* Mill. and *Psidium guajava* (L.); ferulic acid and isoferulic acid were identified in some species, except *Annona cherimola* Mill., *Colocasia esculenta* (L.) Schott, and *Psidium guajava* (L.). Rosmarinic acid and salvianolic acid B were only identified in *Eriobotrya japonica*. (iii) Flavanols, such as catechin and epicatechin, were identified in *Annona cherimola* Mill., *Ipomoea batata* (L.) Poir., and *Psidium guajava* (L.), with a broader distribution; proanthocyanidins, such as prodelphinidins and procyanidins (dimers, trimers, and tetramers), were only identified in *Psidium guajava* (L.). (iv) Flavonols, such as quercetin and its derivatives (rutinoside, glycoside, rhamnoside, etc.), were highly identified in all species. Kaempferol and its derivatives were identified in multiple plants, except *Colocasia esculenta* (L.) Schott and *Psidium guajava* (L.). Myricetin was identified in *Cymbopogon citratus* and *Smallanthus sonchifolius*, while its derivatives were identified in *Psidium guajava* (L.). Isorhamnetin was only identified in *Annona cherimola* Mill. and *Ipomoea batata* (L.) Poir. (v) Flavones, such as luteolin and its derivatives (glycoside and pentoside), were widely identified in all plants, as well as apigenin, which was identified in some plants, such as *Annona cherimola* Mill., *Colocasia esculenta* (L.) Schott, and *Eriobotrya japonica*; chrysoeriol was found in *Colocasia esculenta* (L.) Schott and *Eriobotrya japonica*. (vi) Hydrolysable tannins, such as the HHDP-glucose isomer, vescalagin, castalagin, pedunculagin, casuarina, and tellimagradin I, were identified in *Psidium guajava* (L.). (vii) Alkaloids, such as anonaine, asimilobine, liriodenine, lanuginosine, and pronuciferine, were widely distributed in *Annona cherimola* Mill.

Analyzing Table 2, it is clear that the phenolic compounds present in the above mentioned plant species are vast and fall within different classes and subclasses. Among them, flavonoids and phenolic acids are dominant.

**Table 2:** Bioactive compounds identified in the leaves of selected plants.

Class of Compounds		*Annona cherimola* Mill.	*Ipomoea batata* (L.) Poir.	*Colocasia esculenta* (L.) Schott	*Eriobotrya japonica*	*Cymbopogon* *citratus*	*Psidium guajava* (L.)	*Smallanthus sonchifolius*
Hydroxybenzoic acids	Gallic acid					[59]	[60]	[61]
	Benzoic acid					[59]		
Hydroxycinnamic acids	Neochlorogenic acid				[62,63]	[64]		[63]
	Cryptochlorogenic acid				[62]			
	*p*-Coumaric acid			[65]	[63]	[63,64]		[61]
	3-*O*-Coumaroylquinic acid				[62]			
	Chlorogenic acid	[63]	[63]		[62,63]	[63,64]	[63]	[63,66]
	Caffeic acid hexoside I		[67]					
	Caffeic acid glucoside				[62]			
	Caffeic acid		[67]	[65]	[62,63]	[59,63]		[61,63,65]
	Caffeic acid derivative				[62]			
	1-caffeoylquinic acid		[67]					[66]
	Dicaffeoylquinic acid				[62]			
	3-caffeoylquinic acid		[67,68]					
	4-caffeoylquinic acid		[67,68]					
	3,4-di-*O*-caffeoylqunic acid		[63,67,68]			[63]		
	3,5-di-*O*-caffeoylqunic acid		[63,67,68]					[66]
	4,5-di-*O*-caffeoylqunic acid		[67,68]					[66]
	3,4,5-tricaffeoylquinic acid.		[67,68]					
	5-*O*-caffeoylquinic acid			[65]				[66]
	Feruloylquinic acid				[62]	[64]		
	Caftaric acid				[62]			
	Sinapic acid				[62]	[59]		
	Ferulic acid		[63]		[62]	[63]		[61,63]
	Isoferulic acid		[63]			[63]		
	Rosmarinic acid				[62]			
	Salvianolic acid B				[62]			
Flavanol	Catechin	[69]	[63]				[60]	
	Prodelphinidin B Isomer						[60]	
	Procyanidin B Isomer						[60]	
	Procyanidin tetramer						[60]	
	Procyanidin pentamer						[60]	
	Procyanidin trimer Isomer						[60]	
	Gallocatechin						[60]	
	Galloyl-(epi)catechin trimer Isomer						[60]	
	Procyanidin gallate Isomer						[60]	
	Galloyl(epi)catechin-(epi)gallocatechin						[60]	
	Prodelphinidin Dimer Isomer						[60]	
	Epicatechin	[69]						
Flavonols	Quercetin					[59]		[61]
	Quercetin-3-*O*-rutinoside	[63]	[63]		[63]	[63]	[63]	[63]
	quercetin 3-*O*-galactoside		[67,68]					
	Quercetin 3-*O*-rutinoside-7-*O*-glucoside	[69]						
	Quercetin 3-*O*-rutinoside-7-*O*-pentoside	[69]						
	Quercetin-3-*O*-glucoside	[63,69]	[63]	[63]	[62,63]	[63]	[63]	[63]
	Quercetin-3-*O*-rhamnoside	[63,69]	[63]		[63]			[63]
	Quercetin galloylhexoside Isomer						[60]	
	kaempferol					[59]		[61]
	Kaempferol-3-*O*-glucoside	[69]	[68]		[62]			
	Kaempferol 3-*O*-rutinoside				[62]			
	Quercetin-3-*O*-hexoside		[67,68]					
	Quercetin-3-*O*-hexosylhexoside		[67]					
	Quercetin 3-*O*-malonylglucoside				[62]			
	Myricetin					[59]		[61]
	Myricetin hexoside Isomer						[60]	
	Myricetin arabinoside/xylopyranoside Isomer						[60]	
	Isoschaftoside					[64]		
	Rutin							[61,66]
	Isorhamnetin	[63]	[63]					
Flavones	Luteolin-3′,7-di-*O*-glucoside			[65]				
	Luteolin-6-C-glucoside			[65]				
	Luteolin-7-*O*-glucoside	[63]	[68]		[62]	[63]	[63]	[63]
	Luteolin-8-C-glucoside			[65]				
	Luteolin-4-*O*-glucoside	[63]				[63]	[63]	[63]
	Luteolin 7-*O*-neohesperidoside					[64]		
	Luteolin	[69]		[63]	[62,63]	[63]	[63]	[63]
	Luteolin-3-Galactoside-7-Rhamnoside	[69]						
	Luteolin-3-Glucoside-7-Rhamnoside	[69]						
	Apigenin			[63]				
	Apigenin hexoside				[62]			
	Apigenin derivative isomer 1			[63]				
	Apigenin derivative isomer 2			[63]				
	Apigenin derivative isomer 3			[63]				
	Apigenin derivative isomer 4			[63]				
	Isoorientin					[64]		
	Isoorientin 2-*O*-rhamnoside					[64]		
	Chrysoeriol			[65]	[62]			
	Chrysoeriol rutinoside				[62]			
	7-*O*-malonylglucoside				[62]			
	Apigenin acetylhexoside				[62]			
	Apigenin 6-C-glucoside			[65]				
	Apigenin-6-C-glucoside-7-*O*-glucoside			[65]				
	Apigenin 8-C-glucoside	[69]		[65]				
Hydrolysable tannins	HHDP glucose Isomer						[60]	
	Pedunculagin/Casuariin Isomer						[60]	
	Vescalagin/Castalagin Isomer						[60]	
	Tellimagrandin I Isomer						[60]	
	Tellimagrandin I Isomer						[60]	
	Vescalagin						[60]	
	Ellagic acid deoxyhexoside						[60]	
	Casuarinin/Casuarictin Isomer						[60]	
Alkaloids	Anonaine	[69]						
	Asimilobine	[69]						
	Liriodenine	[69]						
	Stepharine	[69]						
	Lanuginosine	[69]						
	Pronuciferine	[69]						

## 5. Anti-Inflammatory and Anti-Obesity Properties of Bioactive Compounds Identified in the Leaves of Plants Under Study

### 5.1. Hydroxybenzoic Acids

Among the hydroxybenzoic acids found in the leaves of the selected plants, gallic acid emerges as a promising agent against different inflammation-associated diseases. It is not only easily extracted from the leaves but amenable to mass production via biological and chemical synthesis [70]. Various experimental models have gradually confirmed the effectiveness of gallic acid; for example, in obese rats on a high-fat diet (HFD) [71]. Moreover, gallic acid prevents an increase in body weight, while it decreases liver and adipose tissue weights. It also decreases serum parameters, such as triglycerides (TAG), phospholipids, total cholesterol (TC), LDL, insulin, and leptin levels that cause hepatic steatosis, as well as oxidative stress by decreasing glutathione disulfide (GSSG), increasing GSH, GPx, glutathione reductase (GRd), and glutathione S-transferase (GST) [72]. It reduces pro-inflammatory cytokine expression and increases that of junctional proteins in mice as well as Caco-2 cells, thus inhibiting inflammation while ameliorating intestinal mucosal damage via ERβ signaling activation under sodium dextran sulfate-induced colitis [73]. Additionally, supplementing can help reduce excessive lipid stores in obese human subjects, i.e., adipose tissue by inhibiting lipogenesis and improving insulin signaling [71], thereby dampening the pro-inflammatory response and oxidative stress [70,71], and reducing inflammatory cytokines, chemokines, adhesion molecules release, and cellular infiltration [70]. However, its anti-inflammatory action is mainly through MAPK and NF-κB signaling.

### 5.2. Hydroxycinnamic Acids

Derivatives of hydroxycinnamic acid have shown anti-inflammatory and anti-obesity properties both in vitro and in vivo [74].

Supplementation with ferulic acid (FA) in rats fed with a high cholesterol and fat diet (HCHF) for 12 weeks to establish a non-alcoholic steatohepatitis (NASH) model, improved liver coefficients, attenuated the increase in TC, triglycerides (TG), and LDL caused by NASH, improved NASH-induced liver pathological damage, and inhibited the progression of liver fibrosis. FA prevented ROS production and the increase in malondialdehyde (MDA) levels, attenuated the decrease in SOD activity, significantly restored IL-1b, IL-6, and TNF-α levels, and inhibited Rho-kinase (ROCK)/NF-kB signaling pathways in the liver [75]. In another study, mice fed a HFD for 13 weeks showed mitigated lipid deposition in the liver and skeletal muscle [76]. In Wistar male rats supplemented with lard, FA significantly inhibited the increase in plasma lipids and glucose, reduced body weight gain, hyperplasia and hypertrophy in abdominal adipose tissues, and inflammation-associated biomarkers [77]. In humans, FA (1000 mg/day) markedly reduced levels of TC, oxidized LDL, TG, oxidative stress biomarkers, inflammatory markers hs-CRP and TNF-α, and increased HDL in hyperlipidemic patients, potentially reducing cardiovascular disease risk factors [78,79].

Supplementation with p-coumaric acid (PCA) in C57BL/6 mice consuming high fat and high sucrose (HFHS) + PCA (50 mg/kg body weight) for 13 weeks improved mitochondrial dysfunction induced by HFHS, restored muscle fiber size by regulating myosin heavy chain, reduced lipid accumulation/lipogenesis in skeletal muscle with AMPK activation, and mitigated sarcopenic resources by muscle ring finger 1/muscle atrophy F-box protein atrogin-1 (MuRF1/MAFbx) regulation, endoplasmic reticulum (ER) stress markers, and the toll-like receptor (TLR)/NF-kB [80]. In rats, at a dose of 100 mg/kg body weight, PCA reduced the effects of monosodium urate crystals (MSU) due to its *in vivo* anti-inflammatory activity, reducing inflammatory cytokine expression. In a rat model of lipopolysaccharide-induced sepsis (LPS), PCA reduced levels of pro-inflammatory cytokines (TNF-α, IL-1β, IL-6) in lungs and liver, and further increased levels of anti-inflammatory cytokines (IL-4, IL-10) when applied in combination with ellagic acid (EA). In streptozotocin-induced diabetic rats, PCA led to normal insulin, vitamins E and C, SOD, and GSH levels, and increased hexokinase activity, as well as messenger ribonucleic acid (mRNA) expression of glucose transporter 2 (GLUT2) in the pancreas [81]. In male C57BL/6 mice, with an HF + PCA diet (4% PCA in diet, HF + PCA), it improved body weight gain, dyslipidemia, and HF-mediated hyperglycemia, reversed adipocyte inflammation, and protected against LPS-induced inflammation and inhibition of browning via mitochondrial activation [82].

Administration of chlorogenic acid (CGA) in db/db diabetic mice improved GT, insulin sensitivity (IS), and lipid metabolism by increasing fatty acid oxidation and TG lipolysis, and reducing TG synthesis and fatty acid transport in the liver. It alleviated hepatic inflammatory response and oxidative stress, improved microbiota diversity, promoted microbiota composition recovery, and restored expression of inflammatory genes, including TNF-α, IL-1β, IL-6, and antioxidant enzymes, including SOD1, SOD2, and GP_X1_ [83]. In obese mice induced by a high-fat diet (37% of calories from fat), CGA significantly reduced body weight, visceral fat mass, plasma levels of leptin and insulin, triglyceride concentrations (in plasma, liver, and heart), and cholesterol (in plasma, fat, and the heart), significantly inhibited fatty acid synthase activities, 3-hydroxy-3-methylglutaryl CoA reductase, and acyl-CoA: cholesterol acyltransferase activities, while increasing fatty acid oxidation activity and peroxisome proliferator-activated receptors in the liver [84,85]. In Sprague–Dawley rats (hypercholesterolemic) fed a high cholesterol diet supplemented with CGA (1 or 10 mg/kg/day p.o.) for 28 days, it markedly altered the increase in total plasma cholesterol and low-density lipoprotein while reducing high-density lipoprotein (HDL) induced by a hypercholesterolemic diet with dose-dependent improvement in atherogenic index and cardiac risk factor, and attenuated lipid depositions in the liver. These effects were also achieved by supplementation with the caffeic acid (CA) [85]. Furthermore, supplementation with this compound in diabetic mice attenuated inflammatory cytokine concentrations, such as IL-1β, IL-6, TNF-α, and MCP-1 [74]. It also reduced atherosclerosis-associated aortic lesions and body weight of mice fed an HFD, as well as the expression of lipogenesis-related proteins (SREBP1, FAS, ACC, and SCD1) in the liver [86].

### 5.3. Flavanols

Extensive experimental evidence from rodent studies and, to a lesser extent, human studies, have demonstrated that flavanols have significant capabilities to mitigate various events underlying the development of cardiometabolic pathologies [87].

Supplementation with epicatechin (EC) in mouse and rat models of obesity and metabolic syndrome induced by HFD showed the ability to attenuate multiple comorbidities associated with obesity. These effects were observed through upregulation of BDNF and the mitigation of obesity-induced metabolic endotoxemia, along with the attenuation of pro-inflammatory responses and oxidative stress in the hippocampus [88,89]. EC reduced markers associated with oxidative stress and inflammation, improved lipid profiles, enhanced vascular reactivity, and protected against electrical dysfunction [90]. Additionally, it mitigated IR and inhibited visceral adipose tissue inflammation resulting from HFD consumption [88,91].

Catechin, when incorporated in green tea and consumed by overweight and obese individuals, reduced waist circumference (WC) by 4.14 cm and TG levels, increased HDL, and resulted in a body weight loss of 3.5 kg [92,93]. In mouse studies, catechin decreased body weight and TNF-α secretion by M1 macrophages [94]. It also reduced inflammatory cytokines, such as IL-1β and TNF-α, in adipose and hepatic tissues, promoted cholesterol and fat excretion, thereby decreasing blood LDL levels in HFD-induced mice, and inhibited increased adipose tissue weight and plasma lipid concentrations in mice subjected to a high-fructose diet [95].

Proanthocyanidin, when supplemented in obese rats fed an HFD, showed promising results by inhibiting pro-inflammatory molecules, such as CRP, IL-6, and TNF-α, and increasing production of the anti-inflammatory cytokine adiponectin in adipocytes [96,97].

### 5.4. Flavones

Flavones are widely distributed in our diet, found in medicinal plants, functional foods, vegetables, and fruits, playing roles in anti-inflammatory, anti-obesogenic, and anticarcinogenic functions. These properties make them valuable in controlling the innate immune system and inflammation [98,99]. Particularly, apigenin has shown significant evidence, both in vitro and in vivo, by reducing and/or delaying the development of metabolic syndrome [100]. This compound has demonstrated promising results in reducing weight, low-grade inflammation, and insulin resistance. In addition, it attenuates the secretion of a number of pro-inflammatory cytokines, which include TNF-α, IL-1β, IL-6, and even IL-10. For example, apigenin reduces the expression of soluble forms of macrophage markers on monocytes, monocyte adhesion, cyclooxygenase-2 capture, and also promotes nitric oxide production. There is a reduction of inducible nitric oxide synthesis (iNOs) as well as prostaglandin–endoperoxide synthase 2 (COX-2) in subjected tissues and cells of HFD-induced obese mice [101,102]. Under the action of this flavonoid, a reduction of oxidative stress, along with inflammation and apoptosis forced by oxidative stress, as well as an improvement of glycolipid metabolic processes were noted [103]. It inhibits the activation of stress-activated kinases inhibitor of kappa B kinase beta (IKKβ) and Jun N-terminal kinase (JNK), blocks their translocation to the nucleus and also prevents the activation of NF-κB [104]. Together with dysbiosis, apigenin also helped improve intestinal barrier damage and metabolic endotoxemia [101].

Similarly, luteolin was able to lower blood glucose levels as well as blood urea nitrogen (BUN) levels caused by inhibiting apoptotic protein expression and activating the phosphoinositide 3-kinase/protein kinase (PI3K/Akt) signaling pathway in streptozotocin-induced diabetic rats [105]. In addition, the improvement of endothelial function, along with vascular oxidative stress and advanced glycation end-product levels were also noted during the later stages of diabetes in the rats [106]. It has also demonstrated significant anti-inflammatory action, both in vitro and in vivo, interacting with JAK/STAT3 and NF-κB pathways, inhibiting endothelial tissue inflammatory pathways, as well as NLRP3 inflammasome assembly [107].

### 5.5. Flavonols

Recent research has found that the intake of flavonols could be useful in preventing oxidative damage and chronic inflammation that follows obesity, in addition to other benefits on human health biochemically and pharmacologically [108]. Quercetin is the most important representative of this subclass. It has been used clinically in the treatment of IR and atherosclerosis by the definition of a therapeutic procedure aimed at the production and expression of pro-inflammatory cytokines and enzymes, particularly by inhibiting the TLR4/NF-κB signaling system. Quercetin also presents anti-inflammatory action in obesity-related inflammatory gut microbiota and modifies IL-6 levels in the serum of obese children [109]. Moreover, it reduces oxidative stress and inflammation in HFD-induced diabetic rats by modulation of AMPK/SIRT1/NF-κB/Nrf2 signaling pathways [110,111]. Quercetin also acts as an anti-inflammatory and reduces cholesterol, cytokines, and enzymes, and improves endothelial dysfunction due to inflammation [112]. Kaempferol, on the other hand, reduces IL-6 levels by inducing IL-1β, TNF-α and NF-κB domination on mRNA [113]. This compound inhibits kinases and transcription factors due to its anti-inflammatory and antioxidant properties. However, while protecting cells, it can also exhibit cytotoxicity associated with ROS production, depending on its concentration [114].

Treatment with isorhamnetin has been shown to reduce elevated serum glucose levels, restore serum insulin levels, and decrease IR. It also increased GSH levels, GLUT4 protein content, and p-AMPK-α in skeletal muscle, reduced LDL, TG, and TC levels, as well as MDA and IL-6, and GSSG in HFD- and streptozotocin-induced diabetic mice [115]. Furthermore, isorhamnetin has shown significant antioxidant and anti-inflammatory effects in acute kidney injury and acute fulminant hepatitis in mice [116].

Administration of myricetin drastically reduced body weight, serum glucose, TG, TC, hepatic lipid accumulation, thiobarbituric acid reactive substances (TBARS), MDA, and inflammation (TNF-α). Moreover, it increased antioxidant enzyme activities, including CAT, SOD, and GPx activities in high-fat diet-fed mice [116,117]. Myricetin also regulates the expression of various pathways, such as Hippo, MAPK, GSK-3β, PI3K/AKT/mTOR, STAT3, TLR, IκB/NF-κB, Nrf2/HO-1, ACE, eNOS/NO, AChE, and BrdU/NeuN, improving immunomodulatory functions, suppressing cytokines, and enhancing cardiac dysfunction [118].

### 5.6. Hydrolysable Tannins

Hydrolysable tannins are compounds formed from ellagic acid (EA) and gallic acid, linked to a sugar core. They act as primary antioxidants (type I, or chain-breaking), scavenging free radicals, as well as secondary antioxidants (type II, or preventive), exerting effects against free radicals [119].

Administration of these compounds in clinical trials with rodents has shown promising results, including reduction in body weight and weight gain, total oxidative stress, and improved nutrient digestibility in rats with polycystic ovary syndrome (PCOS) [120]. Hydrolysable tannins regulate and modulate the expression and activity of cytokines, reducing the production of inflammatory substances and increasing the formation of complexes with other molecules. This effect includes the suppression of IL-6, IL-8, and TNF-α expression, a decrease in ROS and MDA levels, and an enhancement of SOD, CAT, and GSH. Additionally, they inhibit NLRP3 inflammasome activation by blocking NF-κB signaling, thereby suppressing IL-1β secretion in macrophages [121].

Ellagic acid, in particular, has been shown to increase hepatic levels of GSH, SOD, CAT, and GPx, while reducing levels of aspartate aminotransferase (AST), alanine aminotransferase (ALT), alkaline phosphatase (ALP), nitric oxide (NO), protein carbonyl (PC), MDA, TNF-α, and IL-1β in rats with acrylamide-induced hepatotoxicity [122]. It has also been observed to increase insulin secretion and regulate GLUT4, TG, TC, LDL, HDL, and attenuate TNF-α and IL-6. Furthermore, it reduces ROS and MDA levels, improving oxidative stress in related tissues and mitochondrial function [123].

The therapeutic effect of the phenolic compounds mentioned above was approached individually. However, their co-administration can enhance the individual effects, leading to synergistic interactions. These synergies result from improved bioavailability and antioxidant capacity, targeting multiple signaling pathways and modulation of the gut microbiome, which allow for the reduction of dosages employed, thereby minimizing the risk of potential side effects that, in most cases, occur at therapeutic concentrations [124,125]. Furthermore, recommended therapeutic concentrations are an important factor to consider since the pro-oxidant effect of certain compounds at high concentrations largely depends on the intended application. Indeed, when determining optimal dosages, it is crucial to consider the specific matrix, formulation, and target use, including the nature of the compound, its interaction with other components, and the biological environment in which it will be applied.

## 6. Conclusions and Future Trends

Using powerful leaf-derived bioactive compounds to fight chronic inflammation associated with excessive weight highlights the considerable ability of plant-based bioactive compounds for their role in treatment against obesity and obesity-linked chronic inflammation. Being overweight or obese is a global problem due to unhealthy eating prior to a lack of physical activity. Such conditions have been causally linked with chronic inflammation and oxidative stress factors that contribute to metabolic and cardiovascular diseases.

The research underlines the importance of dietary intervention in tackling these issues. It recommends an anti-inflammatory and antioxidant-rich diet with foods like fruit, vegetables, nuts, seeds, and omega-3-rich fish, while limiting pro-inflammatory food groups such as saturated fat, added sugar, and ultra-processed products. The spotlight turns to bioactive compounds in the leaves of several plants, including *Annona cherimola* Mill., *Ipomoea batata* L. Poir., *Colocasia esculenta* L. Schott, *Eriobotrya japonica*, *Cymbopogon citratus*, *Psidium guajava* L., and *Smallanthus sonchifolius*. These leaves contain phenolic acids, flavonoids, carotenoids, and other bioactive compounds with potent antioxidant and anti-inflammatory properties. These bioactive compounds integrated into the diet help relieve chronic inflammation, improve antioxidant defense, and lower the risk of chronic diseases, including cancer, cardiovascular disease, and neurodegenerative disorders. Clinical research has shown that these compounds effectively lower inflammation markers, reduce oxidative stress, and boost overall metabolic health. The review indicates that routinely eating leaves of plants that are rich in bioactive compounds can be crucial dietary management for obesity and its induced associated health risks. In short, tapping into plant leaves’ nutritional and medicinal value represents a natural and promising method to combat obesity and chronic inflammation. Public health strategies need to endorse and promote the consumption of these bioactive-rich foods to foster healthier dietary patterns and improve population health outcomes. This approach not only supports the prevention and management of obesity but contributes to the overall well-being and longevity of the population.
